# Scale-up Approach in CATI Surveys for Estimating the Number of Foreign Body Injuries in the Aero-digestive Tract in Children

**DOI:** 10.3390/ijerph9114056

**Published:** 2012-11-12

**Authors:** Silvia Snidero, Nicola Soriani, Ileana Baldi, Federica Zobec, Paola Berchialla, Dario Gregori

**Affiliations:** 1 Department of Public Health and Microbiology, University of Torino, Torino, 10126, Italy; Email: silvia.snidero@gmail.com (S.S.); paola.berchialla@unito.it (P.B.); 2 Unit of Biostatistics, Epidemiology and Public Health, Department of Cardiac, Thoracic and Vascular Sciences, University of Padova, Padova, 35121, Italy; Email: nicola.soriani@unipd.it (N.S.); ileana.baldi@unipd.it (I.B.); 3 ZETA Research Incorporation, Trieste, 34129, Italy; Email: federica.zobec@zetaresearch.com; 4 Department of Clinical and Biological Sciences, University of Torino, Orbassano, Torino, 10043, Italy; Email: paola.berchialla@unito.it

**Keywords:** population size estimation, foreign body injuries, social network, scale up methods, self-resolved injuries

## Abstract

Foreign body injuries are a well-known threat to children due to the high risk of ingestion of small objects and choking. In order to depict the epidemiological framework of such injuries, data are mostly available for hospitalizations and partially for emergency room visits. The hidden part of the phenomenon consisting of minor self-resolved injuries is still unknown. The purpose of this paper is to provide an estimate of the overall burden of such injuries in children in Italy. Our paper proposes the use of the scale up technique to overcome most of the pitfalls of classical techniques in the estimation of the number of children aged 0–14 that suffered a foreign body injury in 2004. Our results, based on a CATI survey on 1,081 women, show that the estimated number of children under 15 years that incurred in a foreign body injury was 15,829 (95% CI: 14,376–17,282), of these 12,844 were treated in hospital or in emergency department (95% CI: 11,535–14,153). The scale-up method in conjunction with a CATI survey provides a reliable estimate of the size of hard-to-count populations as those of injured children at lower costs with respect to classical sampling schemes.

## 1. Introduction

Foreign body injuries in the upper aero-digestive tract are due to the aspiration/ingestion/insertion of objects and they are one of the leading causes of injury and death in children [1,2]. Difficulties in estimating the impact of this phenomenon arise because of the large sample size required by classical statistical methods and the consequent possible high costs in planning and processing the surveys [3,4]. To overcome the limitations of classical sampling schemes, several non-probabilistic sampling methods like the capture-recapture method, the snowball and the adaptive sampling have been proposed. Nevertheless, such methods are difficult to implement in the area of foreign body injuries, since: (i) injury rates are highly variable from region to region and are not clustered according to some specific socio-demographic groups. If it were possible to recognize homogenous groups of people that present a bigger incidence of foreign body injuries, then it would be more likely to find people who know a number of other subjects with the same characteristics (and then techniques like snowball sampling could be used); (ii) the lack of completeness of injury registries and the poor reliability and coverage of health administrative data sources which makes the capture-recapture techniques very hard to apply, and (iii) the sensitivity of the questions related to injuries about children, in particular when these are directly asked to parents; indeed, quite a relevant reluctance of parents has been observed, in providing information about injuries occurred to their children [5].

In addition, we should notice that incidence estimates of such injuries usually result from the hospital discharge records and from the death certificates. However, since a large number of foreign bodies injury cases are self-resolved (e.g., parents help children to discharge the object) without accessing the health care services, underestimation of the actual incidence rates is likely to result. 

To overcome these drawbacks, our approach proposes the usage of the scale-up methodology [6,7]. Scale-up method is a probabilistic sampling technique that arose in the field of social networks [8,9,10]. A social network is defined as the set of people that each person knows. The basic idea underlying the scale-up methodology consists in estimating the social network sizes for any given individual surveyed [11].

The estimated network size information is combined with the responses to the questions about how many people the respondents know in the population of children who experienced a foreign body injury. In order to estimate the social network size, individual responses are taken into account for a set of subpopulations with known size and then they are scaled up using the sizes of these groups to the general population. 

Contrarily to the other survey techniques, scale-up methodology does not require asking people directly about problems. This presents two main advantages: first, people are more willing to answer to questions that do not touch directly their person, especially when the topic under investigation is a sensitive issue. Secondly, researchers are able to collect information about all respondents’ social network. This is relevant in particular for studying events with low prevalence, since it allows the use of samples of smaller size as compared to other survey techniques.

This work is aimed at applying the scale-up method to the estimate of the magnitude of the foreign body injury phenomenon, including in this both the known part of it (*i.e.*, the referral to the health care system) and the unknown part (*i.e.*, the self-resolved injuries). Our specific goals were: (i) to estimate the number of foreign body injuries in Italy and (ii) to empirically evaluate the correctness and the efficiency of the scale up estimator in a telephone survey setting.

## 2. The Scale-Up and the Social Network Size Estimators

The scale-up method was developed by Bernard and his colleagues in the 1990s [12,13]. We proposed a maximum likelihood estimator [7] that relies on the assumption that the number *m_io_* of children who suffered a foreign body injury known by the *i*-th respondent follows a Binomial distribution:

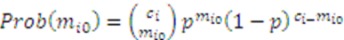
(1)

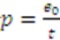
(2)
where *c_i_* is the social network size of the respondent. 

The maximum likelihood scale-up estimator for the size *e*_0_ of the subpopulation *E*_0_ [12,13] is given by:

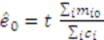
(3)
where the size of the general population *T* is multiplied by the ratio between the total number of children who suffered a foreign body injury known by the respondents and the sum of the respondents’ social network sizes. It has been proven that Equation (3) is an unbiased estimator [13].

The estimator (3) requires just computing the sum of the social network’s size *c_i_* over all respondents. These latter parameters also need to be estimated. Several estimators have been proposed in this regard [8,12,13,14,15,16,17,18,19,20]. In this work, we decided to use the proportional estimator [7,12], which has the same basic underlying idea as the scale-up method, that is, to estimate the social network size of each respondent. People are asked about how many people they know in several subpopulations of known size. Therefore, the proportional estimator for the social network size of the *i*-th respondent is given by the following formula:

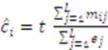
(4)
where *m_ij_* is the number of people known by the *i*-th respondent in the *j*-th subpopulation of known size *e_j_*. As in the scale-up estimator the quantities *c_j_* are assumed known, in this case they are estimated from subpopulations of known size (*i.e.*, the *e_j_* terms are fixed).

### The Selection of Known Size Subpopulations

A sensitive task in the estimation of the social network size is the choice of the subpopulations of known size [19,21]. The basic idea of the scale-up method is that the mean number of people known in a subpopulation is linearly proportional to the size of the same subpopulation [22]. On the other hand, the scale-up estimator relies on the following assumptions: (i) each subject in *T* should have the same probability to know a subject in the subpopulations; (ii) everyone in *T* should know all about his/her acquaintances and (iii) the difficulty to recall in short time all the people known in a certain subpopulation should be assumed to be negligible. It is well known that all these assumptions challenge the linear proportionality relationship [6,22]. 

## 3. Results

**Figure 1:**
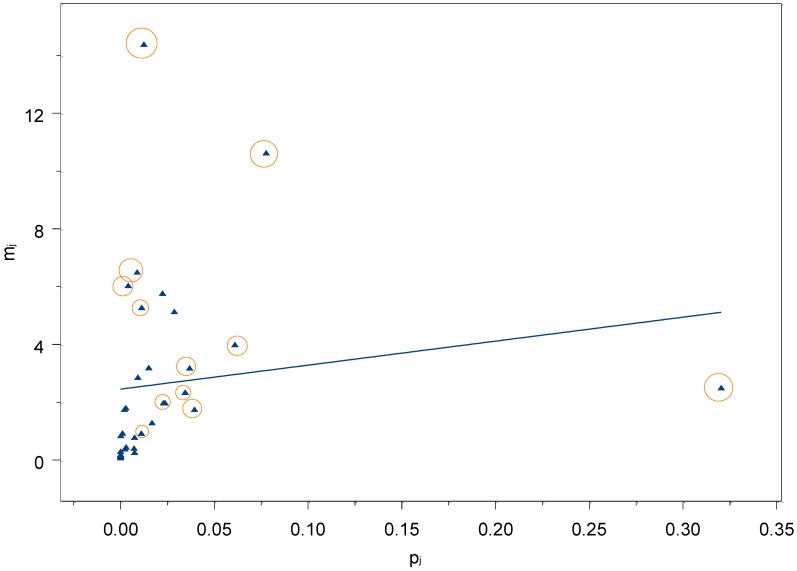
Regression model with all the 33 subpopulations of known size. The circles highlight the subpopulations that were eliminated with the analysis of residuals.

Two-thousand-four-hundred-fifty women have been contacted for the interview, and 1,081 accepted, with a response rate of 44%. Out of a total of 35,673 items, 2,039 answers were not given (5.7%) of which 1,038 referred to the political questions (2.9%). These missing values were replaced imputing the median value of each question. The 1,081 interviewed women recalled a total of 437 children that suffered a foreign body injury, out of which 351 were treated in hospital. The regression model with all the 33 subpopulations (Table 1) accounted just for the 21% of the variability (see Figure 1). 

**Table 1:** Subpopulation of known size used to estimate the respondents’ social network sizes.

Subpopulations of known size	Size in thousands	Subpopulations of known size	Size in thousands
People voted for Casa delle Libertà in 2001 elections	18,300	Widows younger than 60 years	506
Families with 2 children	4,436	People sentenced for driving under the influence of alcohol in 2004	426
People volunteer in non-profit associations	3,481	People went in business during 2004	426
People bought a new car	2,249	Bought a motorcycle	409
Families with only one parent living alone with children	2,101	Competitive basket players members of FIP	169
People voted for Rifondazione Comunista in 2004 elections	1,972	Competitive athletes members of FIDAL	127
Families with 5 or more components	1,635	People doing a temporary job	119
People owing a car with gas or methane	1,356	People currently detained in prison	57
People reported a robbery	1,303	Children adopted in 2004	6
Families with 3 or more children	1,276	People 100 or more years old	6
Own a Mercedes car	956	People committed suicide in 2004	3
People working in hotels and restaurants	859	People reported a rape in 2004	2
Teachers	707	People had a kidney transplantation in 2004	1.7
People with a specialization or a PhD	644	People killed in 2004	0.7
People owing a BMW car	630	People had a heart transplantation in 2004	0.3
Women had a child in 2004	528		

**Figure 2:**
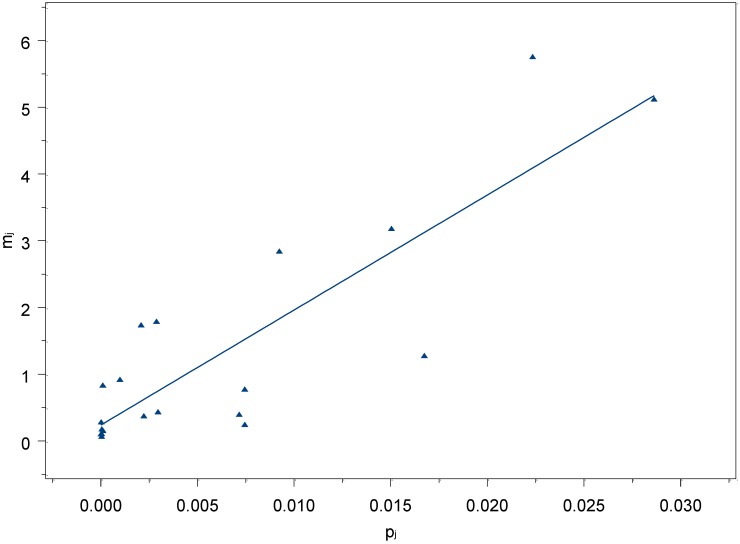
Regression model with the 20 selected subpopulations of known size chosen for the final estimates.

Therefore, we decided to choose the final subgroups to be used in the estimation by assessing the linear relation between the subpopulation sizes and the mean number of people recalled by respondents for each subgroup. Following Snidero *et al.* [7], we performed a visual inspection of the residuals, eliminating those less fitting the linear relationship between subpopulation sizes and mean number recalled by respondents. This lead us to exclude some subpopulations, eliminating at the very end 13 subpopulations and thus obtaining a regression model that accounted for the 79% of the variability (see Figure 2).

As shown in Table 2, this algorithm excluded those subpopulations that are less known by women (e.g., people owing a car with gas or methane) or of bigger size. In the latter case, it is expected that people asked about subpopulations where they know a high number of people, find it difficult to remember the correct number of people that belongs to them (e.g., Teachers, Families with two children, *etc.*). 

**Table 2:** Subpopulations excluded from the analysis using the algorithm based on the regression model.

Excluded subpopulations
People bought a new car
People owing a BMW car
People owing a car with gas or methane
Teachers
Teachers of primary school
People volunteer in non-profit associations
People with a specialization or a Ph.D
Widows younger than 60 years
Families with only one parent living alone with children
Families with 2 children
People voted for Casa delle Libertà in 2001 elections
People voted for Rifondazione Comunista in 2004 elections
People reported a robbery

Using the remaining 20 subpopulations, the estimated mean social network (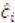) was of 218.3 people and with probability 95%, the interval (142.5–294.2) includes the true value for *c_i_*. This result is not statistically different from a previous estimate of the social network size of an American study that amounted at 286 persons [13]. 

The estimated number of children under 15 years that incurred in a foreign body injury (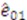) was 15,829 with a 95% CI equal to (14,376–17,282). Out of them, 12,844 cases (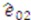) were treated in hospital or in emergency department (95%CI: 11,535–14,153). 

**Table 3:** Results of the sensitivity analysis for each subpopulation excluded from the analysis.

Excl. subpopulation	ĉ	Injury	Hospitalized
Bought a motorcycle	229.0 (40.9)	15,087.6 (706.5)	12,242.1 (636.4)
Own a Mercedes car	232.5 (41.8)	14,862.8 (696.0)	12,059.7 (627.0)
Competitive athletes members of FIDAL	219.2 (39.2)	15,759.2 (738.0)	12,787.0 (664.8)
Competitive basket players members of FIP	220.1 (39.3)	15,699.1 (735.2)	12,738.3 (662.2)
Families with 5 or more components	229.8 (45.3)	15,032.8 (704.0)	12,197.6 (634.1)
People went in business during 2004	225.8 (40.6)	15,302.8 (716.6)	12,416.7 (645.5)
Families with 3 or more children	208.7 (41.4)	16,558.0 (775.4)	13,435.2 (698.5)
People 100 or more years old	217.3 (38.6)	15,902.9 (744.7)	12,903.7 (670.8)
People doing a temporary job	207.3 (38.0)	16,664.0 (780.4)	13,521.2 (702.9)
People working in hotels and restaurants	219.0 (41.3)	15,782.6 (739.1)	12,806.0 (665.8)
People sentenced for driving under the influence of alcohol in 2004	230.5 (41.1)	14,990.1 (702.0)	12,163.0 (632.3)
Children adopted in 2004	211.5 (38.1)	16,333.0 (764.9)	13,252.7 (689.0)
People had a heart transplantation in 2004	217.5 (38.6)	15,886.9 (744.0)	12,890.7 (670.2)
People had a kidney transplantation in 2004	217.5 (38.6)	15,887.2 (744.0)	12,890.9 (670.2)
Women had a child in 2004	211.8 (39.4)	16,315.3 (764.0)	13,238.3 (688.2)
People killed in 2004	216.0 (38.6)	15,996.6 (749.1)	12,979.7 (674.8)
People reported a rape in 2004	217.9 (38.7)	15,858.8 (742.7)	12,867.9 (669.0)
People committed suicide in 2004	217.0 (38.6)	15,924.6 (745.7)	12,921.3 (671.7)
People dead for a cancer in 2004	208.3 (38.1)	16,587.7 (776.8)	13,459.3 (699.7)
People currently detained in prison	212.3 (38.7)	16,270.3 (762.0)	13,201.8 (686.3)

Notes: ĉ is the network size, Injury is the social network size estimate of the number of injuries, for ĉ; Hospitalized is the estimated number of hospitalized injuries; d is standard errors in brackets.

In Table 3 the mean estimates of the social network sizes (*c*) and the target subpopulation size (*e*_0_) obtained omitting one at time of the subpopulations (*e_j_*) are shown. The estimated social network sizes are quite similar for each excluded subpopulation (range: 207.3–232.5) and also the estimates for the number of children that suffered a foreign body injury are in a quite close range (14,863–16,664). This means that the maximum likelihood scale-up estimator and the social network estimator are quite robust to the choice of the subpopulations themselves, in particular when subgroups are first evaluated with a selection procedure to discard those poorly fitting the linearity assumptions of the model. 

## 4. Discussion

Our study estimated that 15,829 children suffered a foreign body injury in Italy in 2004, of which 12,844 were hospitalized or visited at the emergency department. Therefore, the estimated incidence of foreign body injuries in children in Italy is about 0.2%. 

Taking into account that the official number of hospitalized children for such injury was 1,814 in 2003 [23], one child out of nine who incurred in a foreign body injury was hospitalized and one children out of eight who was visited in an emergency department was thereafter hospitalized. These figures are consistent with findings in the injury literature, where a ratio ranging from 1:10 to 1:17 is often observed between hospitalizations and emergency visits [24,25,26]. 

The main limitation is that no extrapolations to any subset of the population are allowed by the method, therefore specific incidence rate such as for any foreign body type cannot be derived by this work.

With the scale-up method we obtained the target information about a number of people virtually equal to the interviewed sample size multiplied by the social network size of each respondent (about 235,000 people). The standard error was quite small (741 persons), corresponding, in relative terms, to 0.9 × 10^−4^ if we divide it by the total number of children aged 0–14 in Italy. In order to getting the same standard error magnitude using the classical probabilistic schemes, we should have planned a sample of exactly 236,254 people. Clearly, the scale up has a multiplying effect on the sample size, due to the indirect contact of a number of people. This is of course particularly appealing in view of rapidity of the CATI survey and of the lower costs associated with it. Indeed, the number of missed items was negligible, and this was obtained while the survey was conducted by interviewers not particularly experienced in the usage of the scale-up technique.

## 5. Methods

### 5.1. Study Design

The study was designed to estimate the number of children aged 0–14 that incurred in a foreign body injury in Italy. The sample was formed randomly selecting 1,081 women aged 18–50 who answered to a CATI survey. The respondents were asked to answer to a questionnaire about:

- The number of people they know in 33 questions about populations of known size;
- The number of children they know that suffered a foreign body injury and some more details related to the injuries (target questions).

The target questions, which were referred to the 2004 year, were:

- “How many children do you know that had an injury due to the swallowing/ingestion of a foreign body?”, aimed at identifying the first target subpopulation of size *e*_01_;
- “Of those, how many children went to the emergency service or were hospitalized?” aimed at identifying the second target subpopulation of size *e*_02_.

Table 1 shows the 33 subpopulations of known size selected from the census and other official sources [27]. Among many definitions of social network given in literature, we adopted that one of “active network”, *i.e.*, “Mutually recognize each other by sight or name, can be contacted, and have had a contact within the last two years, either in person, by phone or mail” [12,13,16]. 

### 5.2. Statistical Analysis

We applied a regression model with the mean number known in each subpopulation as dependent variable and the relative size of the subpopulation as independent variable to select the subpopulations of known size that meet the request of linear proportionality. Therefore, we proceeded to exclude some of the 33 subpopulations with a graphical analysis of residuals. 

According to the formulas given in Equations (1) and (2) we estimated the mean social network size and the target subpopulation size along with 95% confidence interval (95% CI). 

In order to understand how estimates depend on the specific choice of the subpopulations used in the analysis we carried out a sensitivity analysis. A leave-one-out strategy was adopted: one population among the selected subpopulations was picked up and excluded from the sample. This was repeated such that each population was left out once. The analysis was carried out with S-plus ver. 6.2 [28]. 

## 6. Conclusions

The scale-up technique has been shown to have a potential for application in the injury field. A comparison with known figures on foreign bodies injuries has shown a strong consistency with them: of course, since no data exists at the time being on the number of non-hospitalized foreign bodies injuries, the above conclusion is based on common sense and expert opinions. Further data are needed to better understand the hidden epidemiology of such injuries. 

From the methodological point of view, the procedure of selecting sub-populations is still based on the empirical evaluation of the residuals. Less subjective approaches should be investigated. In this sense also the issue of missing data on recalled subpopulation sizes needs further developments; the approach based on the median which has been used in this analysis tends to underestimate standard errors, as shown in some simulation-based investigations in the field of public health research [29], but no data are available for the scale up approach so far. 

Injury research constitutes a terrain where most of the classical techniques can easily fail, due in particular to the high sensitiveness of some questions. In particular, a sensitive problem is how to estimate the number of children who have suffered from injuries or violence at a certain point in their life. This is important public health issue because many children who suffered from injuries often do not go to a hospital or clinic for treatment, in particular in case of facts of lower impact. In addition, children who suffered from violence in their family also find difficulty in reporting the facts. The adaptation of scale up methods to such populations could be a first step toward its use for other public health issues where some cases may be treated outside of the medical system: knowing the size of such populations is important for planning and reducing inequalities in the access of public health care systems. These considerations are emphasized when injuries are related to children and thus when the interviewed parents have to face their sense of inadequacy and guilt. In addition, such technique can successfully be used when official data are not fully recorded or when the data extraction can be costly and time inefficient. In this setting, the possibility to have a reasonable, quick and cost-effective estimation of the magnitude of the phenomenon is surely appealing. 
